# Drivers and solutions to unhealthy food consumption by adolescents in urban slums, Kenya: a qualitative participatory study

**DOI:** 10.1017/S1368980025000400

**Published:** 2025-04-03

**Authors:** Milkah N Wanjohi, Elizabeth Wambui Kimani-Murage, Michelle Holdsworth, Rebecca Pradeilles, Calistus Wilunda, Gershim Asiki, Kerstin Klipstein-Grobusch

**Affiliations:** 1 Julius Global Health, Department of Global Public Health and Bioethics, Julius Center for Health Sciences and Primary Care, University Medical Center Utrecht, Utrecht University, Utrecht, Netherlands; 2 Nutrition and Food Systems Unit, African Population and Health Research Center, Nairobi, Kenya; 3 Montpellier Interdisciplinary Centre on Sustainable Agri-Food Systems (UMR MoISA), University of Montpellier, CIRAD, CIHEAM-IAMM, INRAE, Institute Agro, IRD, Montpellier, France; 4 Chronic Disease Management Unit, African Population and Health Research Center, Nairobi, Kenya; 5 Division of Epidemiology and Biostatistics, School of Public Health, Faculty of Health Sciences, University of the Witwatersrand, Johannesburg, South Africa

**Keywords:** Ultra-processed foods, Adolescent, Unhealthy food, Dietary behaviours, Urban, Photovoice, Participatory research, Sub-Saharan Africa

## Abstract

**Objective:**

To explore the perceptions, drivers and potential solutions to the consumption of unhealthy, ultra-processed foods (UPF) and foods high in fat, salt and sugar (HFSS) and their contribution to the double burden of malnutrition among adolescents living in urban slums, Kenya.

**Design:**

Qualitative participatory research, through Photovoice, group discussions and community dialogues. Inductive, thematic analysis was undertaken.

**Setting:**

Three major slums, Nairobi.

**Participants:**

Adolescents 10–19 years (*n* 102: 51 boys, 51 girls) and adults (*n* 62).

**Results:**

UPF/HFSS consumption emerged as a predominant theme on foods commonly consumed by adolescents, and the causes of undernutrition and overweight/obesity. Adolescents described UPF/HFSS as junk, oily, sugary or foods with chemicals and associated UPF/HFSS consumption with undernutrition, obesity and non-communicable diseases. They perceived UPF/HFSS as modern, urban, classy and appealing to young people and minimally processed foods as boring and primitive, for older people, and those in rural areas. Individual-level drivers of UPF/HFSS consumption were organoleptic attributes (taste/aroma), body size/shape, illicit drug use, convenience and adolescents’ autonomy. Social environment drivers were peer pressure and social status/aspirations. Physical environment drivers were UPF/HFSS availability and accessibility in the slums. Education on healthy eating and the adverse effects of consuming UPF/HFSS, through existing structures (youth groups, school, community health strategy), was proposed as a potential solution to UPF/HFSS consumption.

**Conclusion:**

UPF/HFSS were perceived as associated with poor nutrition and health, yet were preferred over unprocessed/minimally processed foods. Interventions to promote healthy diets beyond raising awareness are important, while addressing the underlying perceptions and drivers of UPF/HFSS consumption at the individual level and in the social and physical food environments.

Optimal nutrition is important for adolescents’ health, growth and development, with recent calls globally to address malnutrition in all its forms^(1)^. Adolescence is a stage of transition and emancipation from dependency on adults to autonomous dietary behaviour^(2)^. As such, it presents an opportunity to establish a foundation of good health and dietary behaviour^(1)^. Healthy behaviour, including dietary choices cultivated during adolescence, can help to achieve optimal nutrition and lower the risks of diet-related non-communicable diseases in adulthood^(3)^. Adolescents’ dietary behaviours are largely influenced by individual factors and the physical and social environments around them^(4)^. The food environment within which they live and interact plays an important role in shaping their food purchase and consumption behaviour^(5)^.

Modern and urban food environments are linked to nutrition transition, characterised by abundant availability, accessibility and promotion of ultra-processed foods (UPF) and foods high in fat, salt and sugar (HFSS), especially in sub-Saharan Africa^(1,2)^. UPF and HFSS (UPF/HFSS) are associated with poor diet quality, including a high intake of saturated fat, salt and sugar, and a low intake of fibre, protein, vitamins^(6)^, fruit and vegetables^(7)^. UPF/HFSS consumption is associated with overweight/obesity, metabolic syndrome and the double burden of malnutrition in adolescence^(8–10)^. Children and adolescents are more likely to consume UPF/HFSS compared with older populations^(11)^. Urban lifestyles, such as eating out, coupled with intensive marketing and advertisement of UPF/HFSS through mainstream and digital media are implicated in the increased exposure and consumption of UPF/HFSS by children and adolescents^(12)^. Additionally, high consumption of UPF/HFSS is documented among socio-economically disadvantaged and food-insecure households^(13)^.

Kenya is experiencing an increase in overweight/obesity among children and adolescents, alongside the persistent burden of undernutrition and unhealthy dietary practices. The prevalence of overweight/obesity among girls has increased from 8 % in 2003 to 13 % in 2022, while 43 % of boys are underweight^(14)^. Unhealthy dietary patterns among adolescents are also documented in urban areas^(15)^. About 70 % of school children and adolescents in Nairobi consume unhealthy foods including sugar-sweetened beverages and ‘junk’ foods^(16)^.

There are ongoing efforts by the government to develop policies to create a healthy food environment to address the burden of malnutrition and diet-related non-communicable diseases^(17)^, but there is a dearth of context-specific evidence on the consumption and drivers of unhealthy foods among adolescents to inform such policies and interventions. As adolescents’ perceptions of factors influencing their dietary behaviours may differ from those of adults, there is a need for more evidence on their perspectives, with global calls for the representation of adolescent voices in the discourse on drivers of food choices and active participation in actions to support healthy dietary behaviour^(2)^. Furthermore, in food environment-related research, the need for qualitative studies and opportunities for learning from people’s perspectives and experiences has been emphasised^(5,18)^. Hence, this study used qualitative participatory research methods, including participatory photography (Photovoice) complemented by focus group discussions and community dialogues, to explore the perceptions, drivers and potential solutions surrounding the consumption of UPF/HFSS and their contribution to the double burden of malnutrition, among adolescents living in Nairobi slums, Kenya. Photovoice is a participatory and visual research methodology introduced by Wang et al.^(19)^. This methodology uses the support of photographs taken by local people to talk about their environment. The photographs then act as a visual prompt for participants, providing them with an opportunity to describe their realities, communicate perspectives and raise awareness of complex (public health) issues in their environments^(20)^. In addition, Photovoice allows researchers to see ‘through the eyes’ of study participants and communities, while the visual products of Photovoice act as effective tools for community dialogues, dissemination, policy engagement and advocacy for social change^(21)^. Photovoice has been used in low-income countries to understand food choices and environments as perceived by adolescents and youth^(22,23)^.

## Materials and methods

### Study site

Data were collected in three of the largest urban slums in Nairobi, Kenya, namely: *Mathare, Korogocho* and *Viwandani*. They are all located in the vicinity of the central business district of Nairobi at an average distance of 5 km (Mathare), 7 km (Viwandani) and 12 km (Korogocho). *Mathare* is the largest of these and one of the oldest slums in Kenya, with an estimated population density of 68 941 persons/km^2(24)^. *Viwandani* is the smallest of these, located in Nairobi’s major industrial area, with an estimated population density of 8554 persons/km^2(25)^. Korogocho is located close to the ‘light industry’ area comprising small/medium factories and artisanal industries, with an estimated population density of 42 401 persons/km^2(25)^. The three slums are generally characterised by high unemployment rates, high poverty and food insecurity, poor housing and congestion; inadequate health, education, water and sanitation infrastructure and high levels of violence, crime and insecurity^(25,26)^.

### Study design

This was a cross-sectional descriptive study, employing qualitative participatory research methods including photovoice, focus group discussions and community dialogues. In Photovoice methodology, participants are provided with a camera that enables them to identify, reflect upon and take photographs representing their experiences and perspectives on issues relevant to the topic of inquiry that affect them^(19)^. The photovoice activities in this study were guided by the format proposed by Wang & Burris^(19)^. It involves participants working in groups to take and discuss photographs representing the issues under research and thereafter organising a community dialogue to exhibit the photographs and stories from the photovoice exercise for subsequent discussion with the community members. This study is part of a larger study focusing on exploring adolescents’ experiences and perspectives on nutrition and malnutrition in urban slums. As such, photovoice and focus group discussions were conducted with older (15–19 years) and younger (10–14 years) adolescents respectively, exploring their perspectives and experiences with (i) undernutrition, (ii) overweight/obesity and (iii) common foods consumed by adolescents. Community dialogues were thereafter organised with community representatives (adults) to discuss the photographs and issues identified by the adolescents on the three topics.

### Sampling and data collection

#### Sampling and sample size

Sampling of adolescents for participation in photovoice and focus group discussions involved purposive quota sampling considering sex (boys and girls) and age (younger (10–14 years) and older (15–19 years) adolescents). As slums are organised in villages (thirteen in Mathare, eight in Korogocho and six in Viwandani), at least four adolescents were selected from each village including a younger boy, a younger girl, an older boy and an older girl. This yielded 108 adolescents from the three slums (*n* 52 Mathare; *n* 32 Korogocho; *n* 24 Viwandani). In order to obtain a wide range and variety of perspectives and experiences in the community, adolescents with varied characteristics such as ethnicity and religious groups were selected for participation in the study.

For the community dialogues, purposive convenience sampling was applied to identify community members representing relevant community groups namely: parents, teachers, health workers, community health promoters and community leaders (chief, village elders, community-based organisation, youth group, women group and religious leaders). Two representatives from the above-mentioned community groups were selected in each slum, yielding a total sample of twenty participants per slum. A sampling strategy indicating the number of participants in each slum is summarised in online Supplementary File 1.

Identification and mobilisation of eligible participants was conducted with support from community health promoters who are part of Kenya’s community health services^(27)^. The community health promoters conduct monthly visits to households to provide basic health information, screening and referrals to health facilities as part of their mandate. They are therefore well informed about households with eligible adolescents and the various community group representatives for the community dialogues.

### Data collection

Data were collected between April and October 2023. The Photovoice and focus group discussions were conducted separately for boys and girls in each slum to facilitate the free expression of opinions without fear of contradiction from the opposite sex. Prior to the main data collection, a pilot exercise was conducted with the adolescents, upon which the photovoice prompt and focus group discussion guiding questions were reviewed and translated into Swahili. The main revisions made involved the translation of technical terms into a language that was easier to understand. This included ‘undernutrition’ into ‘*poor nutrition’* and ‘overweight/obesity ‘into ‘*excessive weight’* as proposed by the adolescents. In addition, due to the high level of insecurity in the slums, concerns were raised about the safety of the younger adolescents walking around the neighbourhood with cameras for the Photovoice exercise. Hence, a decision was made to engage the younger adolescents in focus group discussions only (and not Photovoice) for safety reasons.

#### Photovoice with older adolescents

The Photovoice exercise was undertaken over 4 days: 1 day of Photovoice training, 2 days of photograph taking and 1 day of photograph selection, printing and discussion. On the Photovoice training day, participants were taken through the: (i) consent process, (ii) Photovoice methodology, (iii) use of a camera to take different types of photographs and (iv) ethics of photography, including the no-face or identification details’ protocol to ensure anonymity of people or places^(28)^. Subsequently, participants were provided with digital cameras (Canon IXUS 155) and requested to take at least five photographs that best-represented something (thing or person) in their community that: (i) causes undernutrition (*poor nutrition*); (ii) causes overweight/obesity (*excessive weight*); (iii) should be done to prevent undernutrition; (iv) should be done to prevent overweight/obesity, among teenagers in their community, and (v) food that teenagers commonly eat in their community. The five prompts were generated from the broader study objective of exploring adolescents’ experiences and perspectives on nutrition (food commonly consumed by adolescents) and malnutrition (undernutrition and overweight/obesity) in urban slums.

During the photo-taking exercise, participants worked in small groups to identify and capture photographs that represented the issues that they wanted to highlight. Working in smaller groups enhanced efficiency, engagement and participation of the adolescents in the activities. On the photograph selection, printing and discussion day, the adolescents reconvened into the larger group to review the photographs taken and select those that best represented the stories that they wanted to share. They were then provided with portable printers (Canon Selphy CP 100) and materials (manila paper, pens, glue) to print the photographs, create posters and provide short captions describing the story represented in each photograph. Thereafter, discussions were conducted with the larger group, during which the participants presented and discussed the ‘stories’ of the photographs they had selected.

#### Group discussion with younger adolescents

During the focus group discussions, the adolescents were first organised into smaller groups of at least three participants and asked to reflect and brainstorm on the same five prompts (questions) that the older adolescents were asked to take photographs of namely: (i) *what are causes of undernutrition (poor nutrition);* (ii) *what are causes of overweight/obesity* (excessive weight); iii) *what should be done to prevent undernutrition* (iv) *what should be done to prevent overweight/obesity, among teenagers in this community* and (v) *what foods do teenagers commonly eat in this community*. Thereafter, they reconvened for the main focus group discussion where they presented and discussed the issues they had identified.

#### Community dialogues with community representatives (adults)

One community dialogue was conducted in each slum. This involved a 1-day exhibition of the photographs/posters and issues identified by the adolescents during the Photovoice and focus group discussions, followed by discussions with the community representatives. In the exhibition, photographs were displayed for viewing by the community members, with the adolescents present to provide clarification or explanation on the photographs. They later convened for a discussion, to share their perspective and opinions on the issues highlighted by the adolescents. This step also served as a part of the participant checking and evidence validation process for this study.

All discussions (Photovoice, focus group discussions and community dialogues) were moderated by the first author (M.W.) and an assistant moderator, while probing to obtain more details or clarity where relevant. Both were research officers, had a Master’s level education in nutrition and dietetics and extensive experience in research and qualitative interviewing skills. The discussions were conducted mainly in Swahili, the local language in the study areas. Sometimes participants used local slang, in which case clarification was sought in Swahili. The Photovoice prompts and focus group discussion guides were also translated into Swahili.

All discussions were digitally recorded using an audio recorder and field notes taken during the data collection process. The discussions lasted between 90 and 120 min. The research activities were carried out in venues within the community, which were considered safe and easily accessible by participants. These included the community resource centre in Mathare, a church in Viwandani and a school in Korogocho.

### Data analysis

All discussions were translated and transcribed verbatim in English by a professional Swahili-English transcriber. M.W. reviewed the transcripts for accuracy and coded them in NVivo version 11. Inductive (data-driven) thematic analysis^(29,30)^ was undertaken in the development of the codebook and subsequent coding and analysis. The process involved the identification of ideas, concepts and patterns emerging from the discussions and narratives with the adolescents and community members and organising them into broad themes and subthemes. Data coding and analysis were conducted in three steps. First, the first author (M.W.) read 25 % of the transcripts (*n* 4) and developed the first codebook. This initial codebook was then applied in the coding of all the transcripts, while continually expanding and refining it to include new themes and subthemes generated from the data, with review and contribution by the co-authors. During the coding and analysis processes, consumption of unhealthy foods (UPF/HFSS) emerged as one of the predominant themes, which is the focus of this manuscript. The theme of UPF/HFSS consumption comprised three main subthemes: (i) general perceptions of UPF/HFSS, (ii) the perceived drivers of UPF/HFSS consumption, and (iii) recommendations to address UPF/HFSS consumption by adolescents. The subtheme on perceived drivers of UPF/HFSS was further organised into four categories including individual, social and physical and macro-level drivers, based on the existing conceptual model of adolescent’s eating behaviours^(4)^. Upon the completion of data coding, a matrix was created with the final nodes (themes and subthemes), photographs and excerpts, organised by study slums (Korogocho, Viwandani and Mathare) and type of interview (older boys/girls, younger boys/girls), for comparisons and further synthesis, such as the differences or similarities across slums, sex and age groups.

## Results

In total, 102 adolescents (10–19 years) participated in the Photovoice and focus group discussions and sixty-two adults (≥ 20 years) in the community dialogues (Table 1).

**Table 1.** Characteristics of participants

		Photovoice/focus group discussions (*n*=102)	Community dialogues (*n*=62)
Site (name of slum area)			
	Korogocho	35	19
	Viwandani	35	19
	Mathare	32	24
Age (years)			
	10–14	48	N/A
	15–19	54	14
	20–29	N/A	12
	30–39	N/A	18
	40–49	N/A	10
	≥ 50	N/A	8
Sex			
	Female	51	29
	Male	51	33

Narratives on the theme of UPF/HFSS consumption were organised into three major sub-themes: (i) perceptions of UPF/HFSS, (ii) drivers of UPF/HFSS consumption and (iii) recommendations to address UPF/HFSS consumption by adolescents.

### Perceptions of ultra-processed food/high in fat, salt and sugar

#### Ultra-processed food/high in fat, salt and sugar are sugary, fatty, junk foods and food with chemicals

UPF/HFSS were mainly described as ‘sweet or sugary foods’, ‘chemical foods’, ‘junk foods’ or ‘fatty foods’. They were also described as soft or light foods, which provided less satiety and foods with ‘poor nutrition’ referring to poor nutritional quality. These descriptions were often provided in comparison with minimally processed foods, which were considered as ‘natural foods’, ‘home food’ and ‘strong food’ that provided satiety and better nutritional quality (Table 2).

**Table 2.**
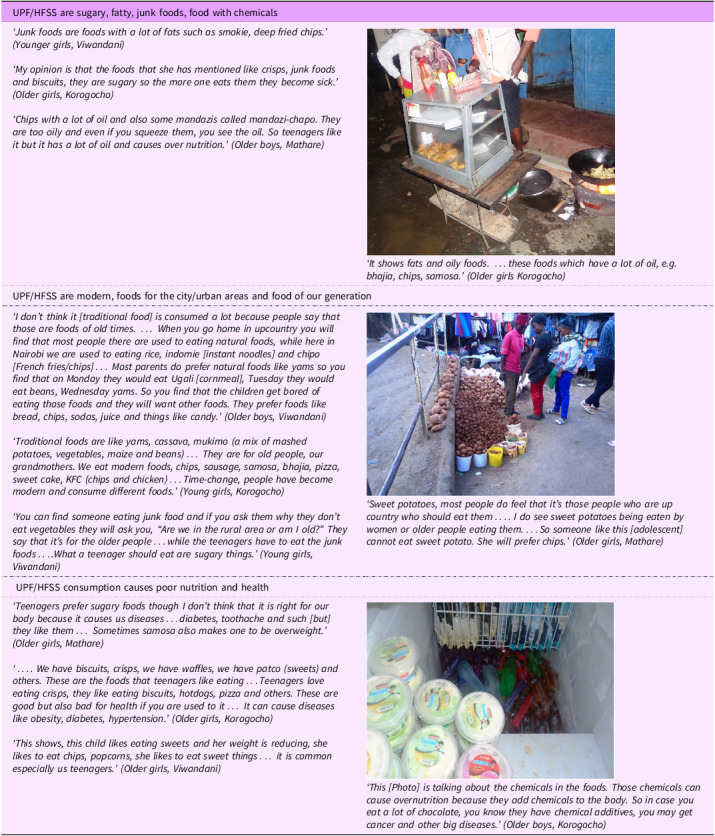
Narratives and photographs on the theme ‘perceptions of UPF/HFSS’

(UPF/HFSS: Ultra processed foods and foods high in fat, salt and sugar; MPFs: unprocessed/minimally processed foods).

#### Ultra-processed food/high in fat, salt and sugar are modern, food for the city and food of our generation

Generational (old *v*. young) and urbanisation (rural *v*. urban) related perceptions on UPF/HFSS consumption were observed. In most discussions, UPF/HFSS were considered as modern foods, fitting for the current generation and for those living in urban areas. In contrast, traditional or minimally processed foods were considered as foods for the older generation, i.e. ‘our parents’ or ‘our grandparents’ or those living in rural areas. Furthermore, UPF/HFSS foods were perceived as exciting and aspirational, but minimally processed foods were in some groups considered as normal or usual, ‘boring’, home food and less appealing to the adolescents (Table 2).

#### Ultra-processed food/high in fat, salt and sugar consumption causes poor nutrition and health

Consumption of UPF/HFSS was perceived as a cause for both overweight/obesity and undernutrition. Participants discussed the sugary/sweet and fatty nature of UPF/HFSS as a cause of overweight/obesity and their poor nutritional quality as a cause of undernutrition. Furthermore, UPF were reported to have chemicals (not specified) and sugar that could cause dental caries and non-communicable diseases, such as hypertension, cancer and diabetes. In some of the discussions, only excessive (not moderate) consumption of UPF was seen as the cause of these diseases. Narratives on UPF/HFSS as a cause of obesity and non-communicable diseases were more widespread than as a cause of undernutrition. The discussions mostly depicted a general knowledge of the link between UPF/HFSS food consumption and poor health outcomes, but there were also indications of knowledge deficits of the unhealthy nature of UPF/HFSS by some adolescents (Table 2).

### Drivers of ultra-processed food/high in fat, salt and sugar

**Figure 1.**
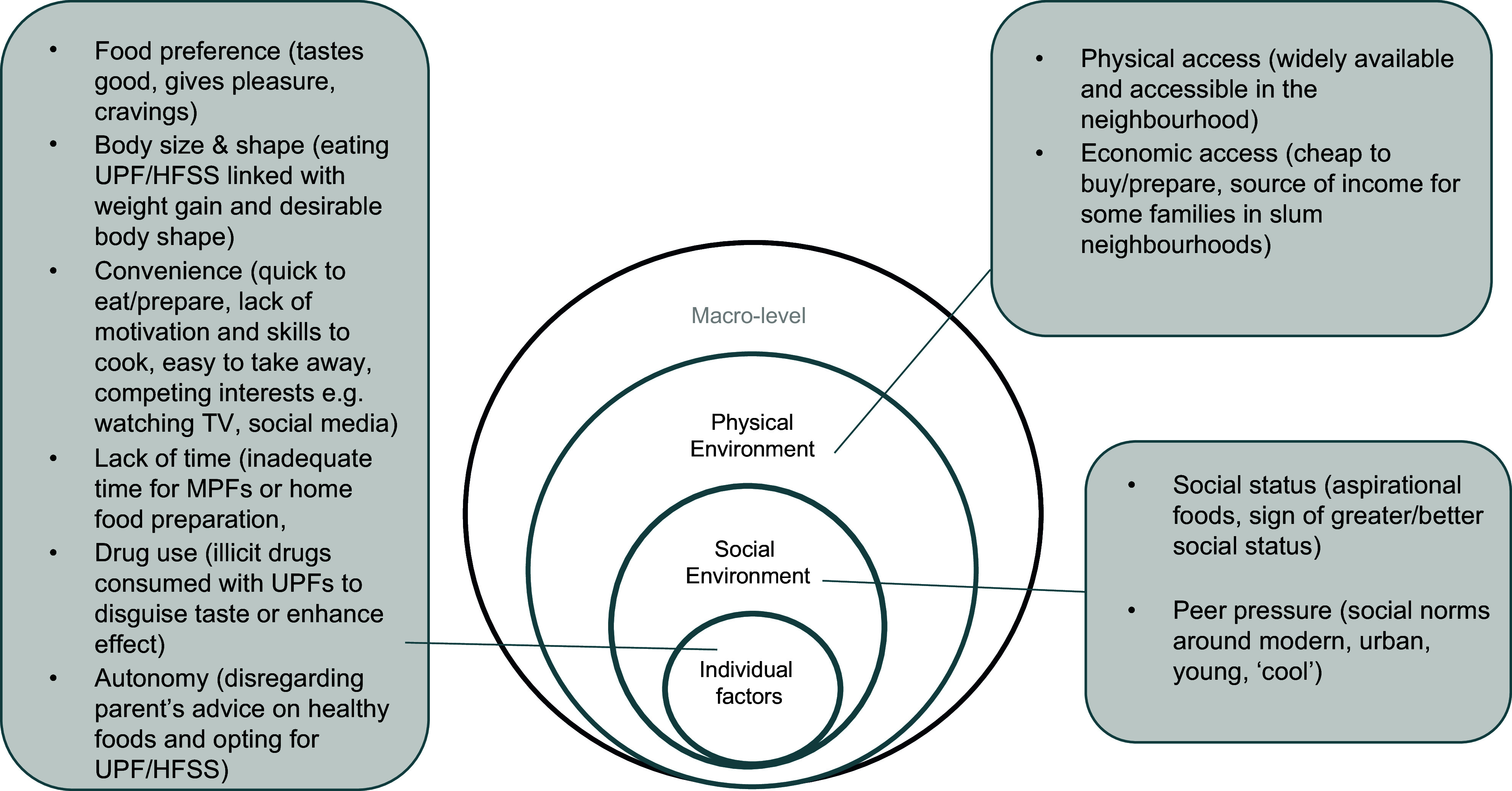
Drivers of UPF/HFSS consumption in adolescents emerging from discussions with adolescents and community members. (UPF/HFSS: ultra-processed foods and foods high in fat, salt and sugar; MPF: unprocessed/minimally processed foods).

Figure 1 presents a summary of the individual, social and physical environmental drivers of UPF/HFSS consumption among adolescents based on the conceptual model of drivers of adolescent eating behaviours. No major themes emerged on drivers of UPF/HFSS in the macro-level food environment.

#### Individual level drivers of ultra-processed food/high in fat, salt and sugar

##### Food preference and sensory perceptions

In most discussions, adolescents acknowledged that they liked and preferred UPF/HFSS compared with minimally processed foods. The main reason for the preference for UPF/HFSS was the perception that they were ‘sweeter’ and tasted better than homemade or ‘natural’ foods. They expressed experiencing cravings and strong desires to consume UPF/HFSS and the satisfaction and pleasure that they experienced with consumption, even with the knowledge that the foods were linked to poor health outcomes. In discussing their preference for UPF/HFSS, comparison was often made with minimally processed ‘natural’ ‘home foods’, which were considered as boring and uninteresting. Participants also reported that home food was monotonous ‘always available’ and easy to access at home, while UPF were not commonly available at home and hence making them more aspirational (Table 3).

**Table 3.**
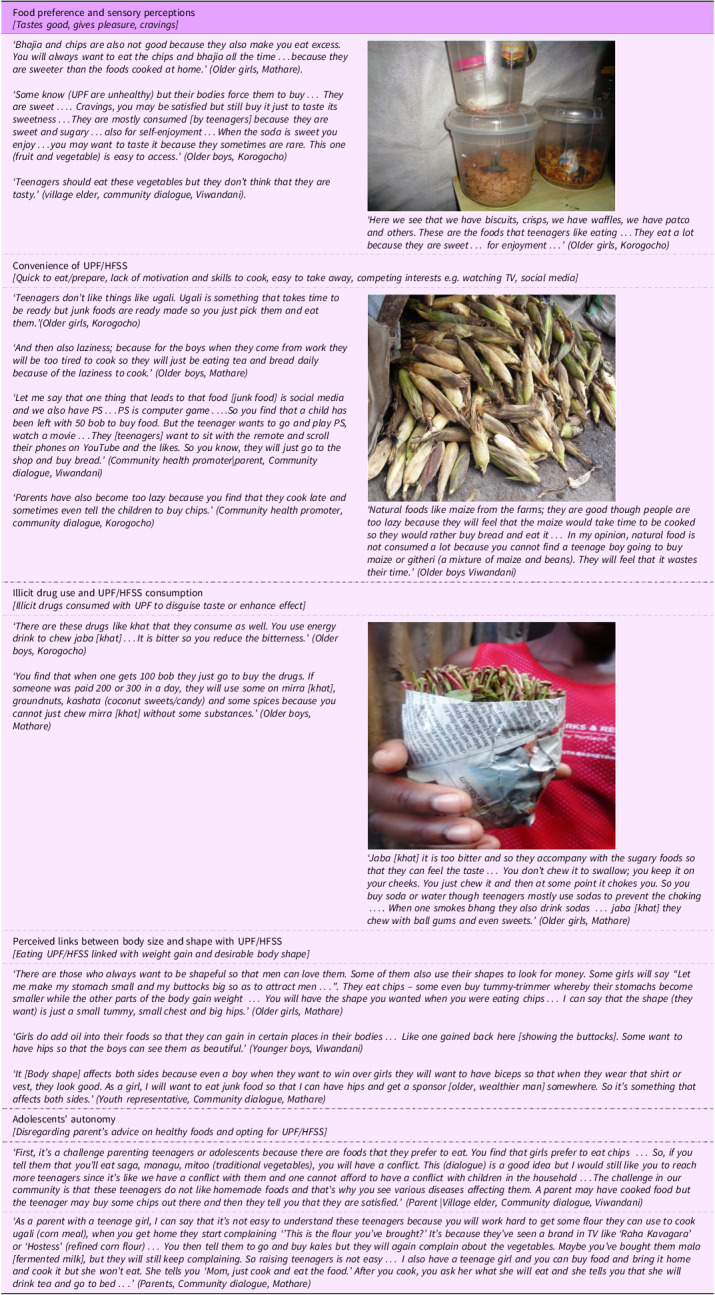
Narratives and photographs on the theme ‘individual drivers of UPF/HFSS’

(UPF/HFSS: Ultra processed foods and foods high in fat, salt and sugar; MPFs: unprocessed/minimally processed foods).

##### Convenience of ultra-processed food/high in fat, salt and sugar

UPF/HFSS were perceived as convenient in comparison to minimally processed foods and home-prepared foods. The perception of UPF/HFSS’s convenience was based on the reason that they (i) were often ready to eat, (ii) required little or no effort for food preparation, (iii) necessitated little or no effort for clean-up thereafter and (iv) were easy to handle, ‘take away’ compared with other foods (e.g. some fruits and vegetables). Discussions on the convenience of UPF/HFSS foods were also reflected in the dialogues with community members. Participants highlighted the common practice of parents giving their children money to purchase ready-to-eat foods from the streets, instead of preparing meals at home, as it was convenient. Some participants also observed that adolescents were often too ‘lazy’ and lacked motivation to prepare meals and would opt for ready-to-eat, often unhealthy UPF/HFSS (Table 3).

##### Inadequate time

Inadequate time was highlighted as among the reasons for preference of UPF/HFSS over ‘natural’ or ‘homemade meals’ that were perceived to require more time for preparation. Adolescents expressed the lack of time to prepare meals at home due to other competing interests, such as work, school and spending time on media such as watching TV or playing video games. The discussion on time deficiency as a driver of UPF/HFSS preference also emerged during community dialogues. Parents highlighted their concerns with adolescents spending too much time on the ‘remote control’ (watching TV) or on social media (‘tiktok’ and ‘You tube’) leaving them with no time to prepare/eat healthy foods. Furthermore, some community health promoters observed that most parents were busy fending for their families and had little or no time to prepare healthy meals at home and hence gave their children money to purchase ready-made food, mainly UPF/HFSS from the streets, without considering the healthiness of the food bought and consumed by their children (Table 3).

##### Illicit drug use longside ultra-processed food/high in fat, salt and sugar

It emerged from the narratives that some UPF, especially sugar-sweetened beverages and candy, were commonly consumed alongside some illicit recreational drugs, such as ‘weed’ (cannabis) and khat. The main reason for this was to amplify the effect of the drugs (weed or khat with energy drinks), mask the unpleasant taste and smell of the drugs (khat with sweetened beverages or chewing gum/candy) or enhance the usability of the drugs (mainly khat and chewing gum). The narratives on UPF and drug use were mainly in Korogocho and Mathare slums and were particularly made in reference to adolescent boys (Table 3).

##### Perceived links between body size and shape with ultra-processed foods/high in fat, salt and sugar

The consumption of UPF/HFSS and mostly those that are fatty/oily was perceived to cause weight gain. As such, some adolescents aspiring to gain weight reported consuming UPF/HFSS to achieve this. In addition, participants described having a body shape with ‘broader hips’ as more attractive to men and peers. Eating fatty and sugary foods was perceived as one of the ways of gaining weight and achieving this body shape. In other discussions, an opposing view emerged, as UPF/HFSS were seen as ‘lighter’ compared with traditional foods and hence preferable for those not wanting to gain weight. Perceptions that UPF/HFSS contribute to weight gain were most prevalent. These discussions on body size and shape preferences mainly referred to adolescent girls (Table 3).

##### Adolescents’ autonomy

Discussion on the autonomy of adolescents in their dietary behaviours emerged from the dialogues with community members and not from the discussions with adolescents. Adolescents were said to be a ‘difficult’ group to deal with, as they did not always listen to their parents’ advice on healthy food preparation and consumption. Some parents highlighted their intention and efforts in preparing healthy meals at home, which the adolescents would reject, opting for unhealthy options such as UPF/HFSS or going without food. They further raised concerns of constant frustrations while trying to encourage and supervise their adolescents’ eating practices and eventually giving up and allowing them to make their own decisions, and on some occasions, following the lead of their children on the foods that they wanted to eat in order to maintain peace in the family (Table 3).

#### Social environment’s drivers of ultra-processed food/high in fat, salt and sugar consumption

##### Social status

UPF/HFSS were perceived as prestigious and ‘classy’ to eat or be seen eating in public. Eating UPF/HFSS was also described by participants as a sign of being progressive and having better economic status in the neighbourhood and at school, compared with ‘ordinary’ home-cooked food or other non-processed foods that would be seen as ‘embarrassing’, ‘primitive’ or a sign of poverty (Table 4).

**Table 4.** Narratives on the theme ‘social environment level drivers of UPF/HFSS consumption’

Social status *[Aspirational foods, sign of greater/better social status]*
*‘She feels embarrassed to eat sweet potatoes but when she eats chips she is proud even if someone sees her. You know girls do love chips.’ (Older girls, Mathare)* *‘You know most girls don’t like being seen to come from poor backgrounds. So they would rather go buy the chips instead of mangoes. In fact you find that maybe bananas are sold at three for ten bob, but people will view you as stupid.’ (Older girls, Viwandani)* *‘Someone will feel that for example instead of buying bananas to eat by the road they would rather buy chips because people will think that you are primitive or such like thing.’ (Older girls, Korogocho)*
Peer pressure to eat UPF/HFSS *[Social norms around UPF/HFSS being modern, urban, young, ‘cool’]*
*‘Some buy ‘junk foods’ because they are influenced by their fellows.’ (Older boys, Korogocho)* *‘… For example at school we have groups of mabombe ‘rich kids’… they will always just buy sodas and go and drink it. So there are maybe some girls whose parents don’t have the money but due to peer pressure she will eat foods that are not beneficial to her every time – soda, crisps and such.’ (Older girls, Viwandani)* *‘Some friends at school just like girls with hips. If you don’t have hips they say that you cannot walk with them. So a friend of mine told a girl ‘you don’t even have hips, go and have some hips before you can come back here’ … Since she had been told in the group that they don’t want someone who is underweight, one will go and buy foods like chipo [chips/French fries], so that she can gain some weight and be loved by the group.’ (Younger girls, Mathare)* *‘So, on balanced diet and especially in school, we find that peer pressure makes the teenagers not eat the traditional foods and all they want are the junk foods. If you cook for them the traditional foods they say that it’s not tasty. So you find that due to peer pressure they want to eat what they want and not what is nutritious.’ (Teacher, community dialogue, Mathare).*

(UPF/HFSS: Ultra processed foods and foods high in fat, salt and sugar; MPFs: unprocessed/minimally processed foods).

##### Peer pressure to eat ultra-processed foods/high in fat, salt and sugar

Peer pressure from friends also emerged as a reason driving the consumption of UPF/HFSS. Participants expressed that they would consume UPF because they saw their friends taking them and would not like to appear different from their friends and schoolmates. In some cases, participants expressed embarrassment from carrying ‘home food’ while their peers had UPF/HFSS. Some community members also expressed their observations on the influence of peer pressure among adolescents in consuming ‘junk foods’ (Table 4).

#### Physical environment level drivers of ultra-processed foods/high in fat, salt and sugar consumption

##### Physical access (availability)

UPF/HFSS were perceived as largely available and accessible in the neighbourhood, sold by both formal (shops) and informal food vendors including roadside (street) food vendors. Photographs taken by participants on UPF/HFSS were mainly of street and informal food vendors, which may be an indication of informal food vendors as a common source of UPF/HFSS in the study area. Although they were readily available in the neighbourhoods, there were perceptions that UPF/HFSS were less common at home, which made them more desirable (Table 5).

**Table 5.**
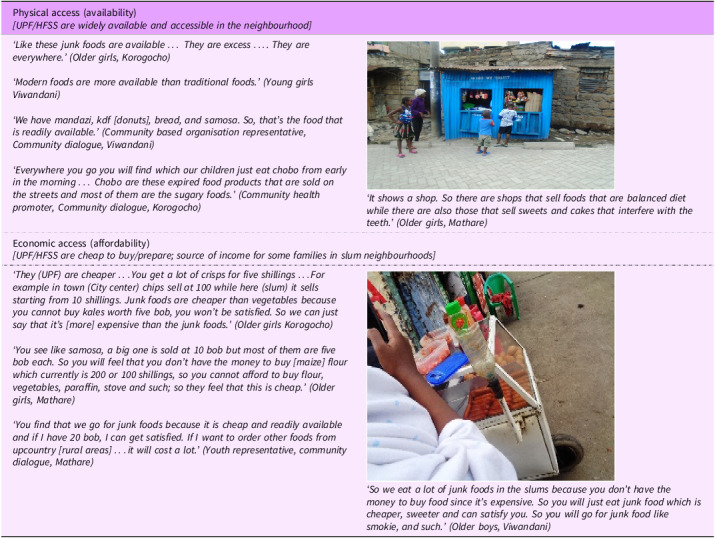
Narratives and photographs on the theme ‘physical environment level drivers of UPF/HFSS consumption’

(UPF/HFSS: Ultra processed foods and foods high in fat, salt and sugar; MPFs: unprocessed/minimally processed foods).

##### Economic access (affordability)

Economic access was discussed in reference to UPF/HFSS food prices in comparison to other non-UPF/HFSS foods, as well as their affordability relative to adolescents’ purchasing ability and the value for money based on the quantity of food that could be bought for a certain amount of money.

Most narratives from adolescents indicated that UPF/HFSS were cheaper in the neighbourhood and available in more affordable quantities compared with minimally processed foods. Additionally, the perception that UPF/HFSS were mostly ready to eat and required less preparation resources, such as fuel and no accompaniments, made them more affordable compared with other homemade foods that needed elaborate preparation and accompaniments, hence requiring extra costs. On some occasions, participants indicated that UPF/HFSS were more affordable in slum neighbourhoods, compared with other parts of the city.

Community discussions supported the perception that some UPF/HFSS were cheaper than non-processed foods and hence were preferred in their community in the context of high food prices and difficult economic situations (Table 5).

### Recommendations to address ultra-processed food/high in fat, salt and sugar consumption

Nutrition education and awareness creation on the unhealthy nature of UPF/HFSS and the negative health effects associated with UPF/HFSS consumption were the most common recommendations by adolescents and community dialogue participants. Existing structures, such as schools, youth groups and community meetings, were recommended as potential avenues for education and awareness creation on healthy diets for adolescents. In addition, the inclusion of adolescent nutrition components in the community health strategy through the community health promoters training modules was recommended, to enable them to reach out to adolescents during their regular monthly household visits.

Banning the sale of UPF in their neighbourhoods was also recommended as a way of curbing their consumption. However, some adolescents had the view that UPF/HFSS were a core part of urban food sources and would therefore be difficult to eliminate. It further emerged from the discussions that UPF/HFSS sales and business were important income-generating activities in the neighbourhood. As such, the adolescents were concerned that eliminating UPF/HFSS would render them or their families economically inactive (Table 6).

**Table 6.**
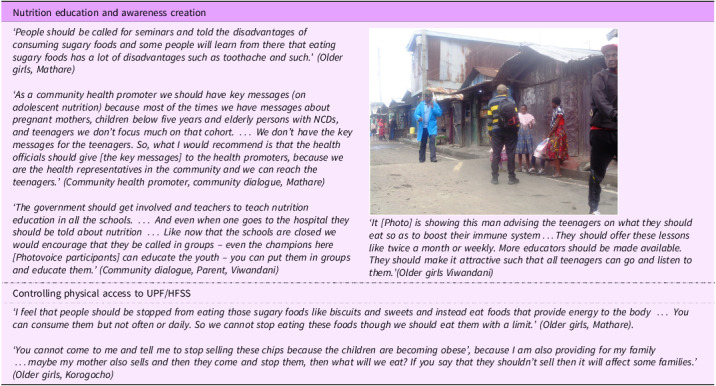
Narratives and photograph on the theme ‘recommendations to address UPF/HFSS consumption’

(UPF/HFSS: Ultra processed foods and foods high in fat, salt and sugar; MPFs: unprocessed/minimally processed foods).

## Discussion

This study sheds light on adolescents’ experiences and perspectives on UPF/HFSS consumption, complemented by the observations of other community members. The findings indicate widespread popularity and positive regard for UPF/HFSS foods compared with non-UPF/HFSS ‘natural’ and ‘homemade’ meals amongst adolescents in urban slums, Kenya. The study further reveals a contrast between adolescents’ knowledge and their preferences or practices regarding UPF/HFSS. There is, a general awareness of the unhealthy nature of UPF/HFSS and their potential contribution to poor nutrition and health outcomes. There is also awareness of the healthiness of unprocessed or minimally processed foods and their association with good health and nutrition. Despite this awareness, there is a greater preference for UPF/HFSS compared with minimally processed foods.

The disconnect between adolescents’ knowledge and preferences (and potential practices) is fuelled by the perceptions that UPF/HFSS foods are modern, urban, classy, a sign of better social status for the young generation and the opposite perception for minimally processed foods. Spending time on media, such as watching TV, gaming and social media emerged as a potential competing interest, deterring them from preparing and consuming healthier homemade meals. A review of worldwide UPF consumption showed that they are indeed more commonly consumed by younger (than older) populations^(11)^ with indications that children and adolescents are their earliest adopters in low- and middle-income countries^(12)^. Other studies indicate that deliberate strategies by UPF industries to market and promote UPF to children and adolescents, such as campaigns in broadcast and social media may influence their attitudes and perceptions^(31,32)^. Strategies to limit the marketing and advertisement of unhealthy foods to children and adolescents, while promoting healthier foods, should be implemented to counter the positive attitudes and preference for unhealthy UPF/HFSS and the widespread perception that minimally processed foods are boring and for older or rural populations.

Factors driving the consumption of UPF/HFSS were identified at the individual level, as well as in the social and physical environment that they live and interact with.

At the individual level, food preferences, largely driven by the food’s organoleptic qualities and convenience influenced the consumption of UPF/HSSF in line with previous findings^(33,34)^, in which out of over 100 possible factors identified, food preference was ranked among the top five drivers of dietary behaviours in the African context^(35)^. UPF by design have high sensory appeal, are highly palatable and often ready to eat, requiring little or no preparation^(36)^. The various processes in the production and preparation of UPF/HFSS such as deep frying, the addition of artificial flavours and colours, emulsifiers, sweeteners, carbonating, gelling and glazing agents, enhance their organoleptic qualities^(36)^. The high preference for UPF/HFSS compared with alternative healthier minimally processed foods is of concern given the increasing literature that links UPF/HFSS consumption with adolescents’ poor diet quality, such as high calorie and poor nutrient intake, and poor health outcomes such as overweight/obesity, micronutrient deficiencies^(10)^ metabolic and cardiovascular diseases^(8,37)^. Interventions to educate and provide practical support to adolescents to prepare recipes from affordable, convenient, appealing (tasty/socially desirable), healthier, minimally processed foods are therefore needed in urban contexts.

Body size and shape emerged as an important issue driving the consumption of UPF/HFSS. Adolescence is a stage where body awareness and romantic relationships begin^(38)^, which could explain the aspiration towards body shapes that are perceived to be attractive. In a previous review of body size preferences for African women, a large body size (but not obese) was seen as a traditional African ideal and associated with strength, wealth and prosperity while a full figure was perceived as sexually attractive^(39)^. Literature on the role of personal values in dietary behaviours indicates that the values or issues that consumers perceive as important to them, may ultimately drive their decisions on food consumption^(40)^. This may explain the preference and choice to consume UPF/HFSS by some adolescents in pursuit of a perceived attractive body shape, despite the knowledge of their unhealthy nature. This therefore calls for interventions to shift social norms about what a healthy weight is and how to achieve it safely through a healthy diet and physical activity, as well as addressing the underlying attitudes that cause stigmatisation of certain bodies among adolescents.

We also observed recreational illicit drug use to be a potential driver for UPF consumption among adolescents in this study, in line with previous results showing the potential link between UPF consumption and illicit drug use among adolescents^(41)^. Further research on this issue, as well as integration of nutritional messages into campaigns against illicit drug abuse by adolescents, is warranted, especially in urban slums and similar contexts where drug and substance use among teenagers especially boys, is prevalent^(42)^.

Within the physical environment, the availability and accessibility of UPF/HFSS as a driver of consumption as highlighted in this study have been documented in a previous study^(43)^. Urbanisation and nutrition transition are implicated in the wide availability of a variety of unhealthy foods and changes in dietary behaviour from traditional diets to highly processed, cheap, energy-dense and nutrient-poor diets such as UPF/HFSS^(44)^. Street and snack foods in urban areas are widely available and documented as a source of readily available, affordable and convenient calories, which drives their consumption by adolescents in such contexts^(5,42,43,45)^. Urban areas are characterised by busy lifestyles, often going along with minimal time for the preparation of healthy homemade meals. Urban slums are further characterised by high levels of food insecurity and poverty^(26)^, a situation that worsened during and soon after the COVID pandemic leading to increasing food prices, especially staple foods (e.g. cereals and legumes) often used for healthier homemade meals^(46)^. Previous research indicates that some of the UPF industries are multinationals, well-financed to ensure that their products remain affordable in the context of economic adversity, including the provision of a variety of low-cost products targeting different market segments ^(43)^. These may be some of the reasons why UPF/HFSS were reported to be more affordable and convenient and cost-effective options compared with minimally processed foods and homemade meals. In line with this, food prices were ranked as one of the most important drivers of dietary behaviours in the African context^(35)^. Strategies such as food price interventions are advocated to improve the availability and affordability of healthier non-UPF/HFSS foods and limit consumption of UPF/HFSS and address the widespread access to UPF/HFSS. Fiscal policies to increase taxes on unhealthy foods and subsidise healthier foods are among the recommended interventions to create a healthier food environment^(47)^.

There was a widespread recommendation from participants that nutrition education was needed to shift attitudes and dietary behaviours. Existing literature further indicates that knowledge alone may not be sufficient to trigger healthy dietary behaviours among adolescents^(2)^. This is partly because health and nutrition outcomes are not a major priority for adolescents’ dietary behaviours but rather the immediate benefits such as convenience, satiety and sensory gratification, provided for example by UPF/HFSS^(48)^. This, according to Neufeld *et al.*, is because the consequences of poor diets are often not experienced immediately, but in adulthood, which may seem too far away to motivate adolescents to change their behaviour^(48)^, indicating the need for multifaceted and multisectoral interventions to promote healthy dietary behaviour among adolescents and address the drivers of UPF/HFSS consumption. Guided by the Behaviour Change Wheel for designing interventions^(49)^, emphasis should be placed on (i) education and training to increase and reinforce the knowledge and understanding of healthy dietary behaviour for optimal health and nutrition and the adverse effects of UPF/HFSS, through schools, community and peer groups and the community health strategy, (ii) modelling behaviour through practical demonstrations to enable them to prepare recipes from affordable, convenient, appealing (tasty/socially desirable) healthier, minimally processed foods, (iii) food environmental restructuring to increase the availability, accessibly of healthier non-UPF/HFSS foods in the urban slum neighbourhoods and (v) incentivisation and empowerment of food vendors to provide healthier and safer non-UPF/HFSS foods in slum neighbourhoods. Policy actions to create a healthy food environment that supports healthy dietary behaviour for adolescents may also focus on (i) regulations and legislation for marketing and promotion of unhealthy foods such as UPH/HFSS targeting children in and around schools and traditional and social media, (ii) fiscal measures to increase taxes on unhealthy foods (e.g. UPF/HFSS) and subsidise healthy non-UPF/HFSS foods, (iii) marketing and communication strategies for promoting healthy foods and dietary behaviour and addressing underlying perceptions (UPF/HFSS are urban, modern, classy, for young people) and misconceptions (e. g. body shape/size social class) that fuel UPF/HFSS preference and consumption by adolescents.

### Strengths and limitations

A particular strength of this study is the participatory research undertaken, empowering adolescents to document, reflect upon and discuss in detail their own perspectives and experiences with UPF/HFSS consumption in their communities, by use of photography and group discussions. Community members added a complementary perspective, which combined with adolescents’ views, allowed for a more holistic understanding of the issues and identification of possible solutions. The photographs generated by participants assisted the researchers to better understand the perspectives and experiences of the adolescents and probe for more in-depth information, hence enriching the insights. A potential limitation is that the photography exercise was only conducted for 2 days. More days would have allowed the adolescents longer time for reflection and photography, but this could not be realised because the majority of participants were school-going children and were only available during the weekend. However, during the discussions, adolescents were encouraged to talk about any other issues that were not addressed in the photographs that they had already taken. Other limitations include potential desirability in reporting the foods commonly consumed by adolescents in the study community; however, this was mitigated through triangulation of data from adolescents of different sex and age groups and the community representatives. Furthermore, the narratives on the causes and solutions to unhealthy food consumption were mainly at the individual, social and physical levels and not at the macro level (see Figure 1).

### Conclusions

This study highlights a general understanding of the adverse nutrition and health outcomes associated with UPF/HFSS consumption by adolescents living in urban slum settings in Kenya. However, this knowledge does not necessarily translate into positive action towards healthier choices, as evidenced by the general preference for UPF/HFSS compared with healthier non-UPF/HFSS, fuelled by perceptions that UPF/HFSS foods are modern, urban, classy foods and for the young generation while minimally processed foods are boring and for older and rural people. Factors at the individual level (e.g. organoleptic food preferences, body shape, illicit drug use, convenience, time deficiency and autonomy), physical environment (UPF/HFSS, physical and economic access) and social environment (social status, peer pressure) influence the consumption of UPF/HFSS. There is a need for multifaceted, multisectoral interventions to promote healthy dietary behaviour among adolescents and address the perceptions and drivers of UPF/HFSS consumption. Adolescents should be involved in the design and co-creation of such interventions, given the role of adolescents’ autonomy in decision-making regarding UPF/HFSS consumption as revealed in this study.

## Supporting information

Wanjohi et al. supplementary material 1 Wanjohi et al. supplementary material

Wanjohi et al. supplementary material 2 Wanjohi et al. supplementary material

## Supplementary material

For supplementary material/s referred to in this article, please visit https://doi.org/10.1017/S1368980025000400
