# Supplementary material for: Drivers and solutions to unhealthy food consumption by adolescents in urban slums, Kenya: a qualitative participatory study
Source: Public Health Nutr. 2025 Apr 3;28(1):e123. doi: 10.1017/S1368980025000400 (PMC12465072; doi:10.1017/S1368980025000400)
Supplement: Wanjohi et al. supplementary material 1 — Wanjohi et al. supplementary material [file S1368980025000400sup001.docx]

**Drivers and solutions to unhealthy food consumption by adolescents in urban slums, Kenya: A qualitative participatory study**

**Supplementary file 1**

Supplementary file 1 : Sampling strategy for the study

|  | Mathare ( 13 villages) | Korogocho ( 8 villages) | Viwandani ( 6 villages) | Total |
| --- | --- | --- | --- | --- |
| Photovoice participants |  |  |  |  |
| *Older girls* | 13 | 8 | 6 | **27** |
| *Older boys* | 13 | 8 | 6 | **27** |
| Focus group discussion participants |  |  |  |  |
| *Younger girls* | 13 | 8 | 6 | **27** |
| *Younger boys* | 13 | 8 | 6 | **27** |
| Total number of adolescents per slum | 52 | 32 | 24 | **108** |
| Community dialogue participants | 20 | 20 | 20 | **60** |
| Total number of participants per slum | 72 | 52 | 44 | **168** |
